# The Effectiveness and Safety of a Skin Care Product With *Centella asiatica* Leaf Extract, Ceramide NP, and Panthenol in Subjects With Sensitive Skin: A Prospective, Observational Study

**DOI:** 10.1111/jocd.70324

**Published:** 2025-07-19

**Authors:** Zhen Su, Yue Zheng, Jinling Yi, Wei Lai, Congxiu Ye

**Affiliations:** ^1^ Department of Dermatology The Third Affiliated Hospital of Sun Yat‐Sen University Guangzhou Guangdong People's Republic of China; ^2^ Department of Dermatology NanFang Hospital, Southern Medical University Guangzhou Guangdong People's Republic of China

**Keywords:** barrier function, cosmetics, facial cream, moisturizer, redness, sensitive skin, skin hydration

## Abstract

**Background:**

Sensitive skin is a hyperreactive condition with rising prevalence. A holistic skin care routine is crucial for management. Selecting appropriate skin care products is of great concern in clinical practice.

**Aims:**

To assess the effectiveness and safety of a facial cream containing *Centella asiatica* leaf extract, ceramide NP, and panthenol in Chinese females with sensitive skin.

**Methods:**

In this single‐center, prospective, self‐controlled study, 88 female participants applied the cream on their whole face, with follow‐ups at Day 2, Weeks 1, 2, and 4. The primary endpoint was the change rate of the SS—10 global score at Week 2. Non‐invasive skin parameters and subject self‐assessment were conducted. Adverse events (AEs) were monitored.

**Results:**

Mean SS‐10 scores dropped by 66% and 76% after 2 and 4 weeks. Four symptoms (skin irritation, tautness, itching, and redness) were significantly reduced. TEWL decreased at Weeks 1 and 2, and skin pH at Week 1 and Weeks 2 and 4. SCH increased at all time points and sebum secretion at Week 4. EI, a* value, and facial red area rapidly decreased. Greater improvements were seen in the sensitive skin group compared with the slightly sensitive skin group. All AEs were mild and self‐limiting.

**Conclusions:**

This study showed the facial cream was effective and safe for 4‐week use. It provided immediate relief of facial redness and discomfort, strengthened skin barrier, and modulated pH and sebum secretion, indicating favorable soothing and barrier‐repairing properties for sensitive skin.

## 1 Introduction

Sensitive skin is a prevalent syndrome of cutaneous hyperreactivity, which is characterized by subjective symptoms such as burning, stinging, itching, and tightness triggered by multifactorial stimuli, and may also be accompanied by objective signs like erythema [1, 2]. Its increasing prevalence is linked to modern lifestyle factors (diet, alcohol, smoking), environmental exposures (pollution, climate, chemicals), or endogenous triggers (psychological stress, hormonal fluctuations, gender differences) [3, 4]. A meta‐analysis published in 2020 showed a high prevalence of sensitive skin globally, with an overall self‐declared sensitive skin population as high as 71% [5]. Although the clinical symptom tendency of sensitive skin varies among different ethnic groups around the world, the mechanism of occurrence is basically the same [6]. In China, a 2019 nationwide survey revealed that 49.6% (*n* = 10 956/22 085) of females self‐identified with sensitive skin—a 25.6% increase from 2013 data [7], highlighting the escalating severity of this condition among Chinese women. Although many hypotheses have been proposed regarding the underlying pathogenesis of sensitive skin, such as impaired barrier function, activation of TRPV channels, etc., further exploration is needed to achieve a better understanding [8, 9]. This also means that there is no “gold standard” for the management of sensitive skin at present. The current strategies are mainly repairing damaged skin barrier and inhibiting neurogenic inflammation [2, 9].

Given that sensitive skin is difficult to quantify, a reasonable assessment method is essential [1]. Commonly used subjective questionnaires include the Dermatology Life Quality Index (DLQI) [10], Sensitive Scale‐10 (SS‐10) [11], Body Skin Discomfort Index (BSDI) [12], and Burden of Sensitive Skin (BoSS) [13, 14]. Among them, the SS‐10 is the first scale developed by Misery et al. and validated in multiple countries for quantifying the severity of skin sensitivity. The scale consists of 10 items scoring one visible skin sign and nine subjective symptoms over the preceding 3 days, with higher scores indicating greater severity [11]. In addition, objective measurements are mainly based on non‐invasive skin physiological parameters, such as transepidermal water loss (TEWL), stratum corneum hydration (SCH), sebum secretion level, and erythema index (EI), etc., which could better reflect sensitive skin conditions and barrier function changes [15, 16].

The complexity of diagnosis and treatment brings great challenges to skin management. At the same time, with the increasing attention to skin care routines, the demand for specialized skincare products for sensitive skin is also growing. The expert consensus on holistic skin care [17] recommends that sensitive skin should be managed with the CTMP (Cleanse, Treat, Moisturize, Protect) procedures. Particularly, moisturizers play a crucial role in overall skin care to improve skin hydration and barrier function [17, 18].

Cetaphil soothing and comforting facial cream has been specially developed for sensitive skin, containing *Centella asiatica* leaf extract, ceramide NP, and panthenol. As daily care products for sensitive skin, the ingredients of moisturizers should be able to improve barrier function and reduce neurogenic inflammation, as well as provide a moisturizing and soothing effect [2, 3, 19]. *Centella asiatica* extract contains asiaticoside and flavonoids, which possess antioxidant, anti‐inflammatory, and moisturizing properties [20, 21]. Ceramide NP forms lamellar barriers within the stratum corneum, crucial for maintaining barrier integrity and water retention [22]. A decrease in ceramide levels can lead to impaired skin barrier function and dryness, while topical application enhances endogenous ceramide production to facilitate repair [22, 23, 24]. Panthenol, a precursor of vitamin B5, can be converted to pantothenic acid upon absorption, which improves skin hydration, reduces TEWL, and alleviates inflammation [21, 25, 26]. Previous human efficacy evaluations have shown that this cream can repair the skin barrier and provide long‐term moisturizing efficacy. In this study, we performed a single‐center, prospective, and self‐controlled study based on a larger sample size to evaluate the efficacy and safety of this facial cream in Chinese females with sensitive skin using a more comprehensive approach.

## 2 Methods

### 2.1 Study Design

This study was a single‐center, prospective, and self‐controlled trial. The study protocol and all amendments were reviewed and approved by the Ethics Committee of the Third Affiliated Hospital, Sun Yat‐sen University. This study adhered to Good Clinical Practice (GCP) guidelines and the Declaration of Helsinki. Written informed consent was obtained from all subjects before the start of the study. Consistent conditions were maintained for both questionnaire assessments and instrumental measurements during each follow‐up visit. The same researcher conducted the evaluations and measurements on the same target area under relatively constant environmental conditions (a room temperature of 21°C ± 1°C and humidity of 50% ± 5%). Before each test, subjects were instructed to avoid strenuous exercise and to rest for 20 min in a stable indoor environment.

### 2.2 Study Subjects

Healthy female subjects aged ≥ 18 years with sensitive skin as judged by a professional dermatologist were selected. The diagnosis of sensitive skin was based on the Chinese expert consensus on the diagnosis and treatment of sensitive skin (2017) [2]. Subjects were prone to subjective symptoms such as burning, tingling, itching, and tightness when their skin was stimulated by physical, chemical, and mental factors, and the lactic acid stinging test (LAST) was positive (a cumulative score of ≥ 3). Subjects were excluded if they were pregnant or lactating, or had used other drugs, cosmetics, and medical aesthetics treatments that had an impact on the study results within 2 weeks before the study.

### 2.3 Test Product and Treatment

The investigated product is a soothing and comforting facial cream containing *Centella asiatica* leaf extract, ceramide NP, and panthenol, which is manufactured by Galderma Canada Inc. All subjects were instructed to apply an appropriate amount of this cream to each side of the face twice daily. Other daily skin care products, such as cleansing products, sunscreen, serum, etc. were kept unchanged during the study.

### 2.4 Evaluation

#### 2.4.1 Sensitive Scale‐10

The primary endpoint was to evaluate the improvement in skin sensitivity, measured by the change in the SS‐10 global score after 2 weeks of cream use. The skin sensitivity was assessed using a 10‐item version of the SS‐10 (0: zero intensity, 10: intolerable intensity) including nine subjective symptoms and one visible skin condition (Figure S1). A higher score means a higher severity [11].

#### 2.4.2 Equipment Measurements

Secondary endpoints were non‐invasive skin physiological parameters, including TEWL, SCH, sebum secretion level, skin pH, erythema index (EI), and redness (a* value, facial red area). Subjects' demographics were recorded at the baseline. Equipment measurements (SS‐10 score, TEWL, SCH, skin pH, EI, a* value, and facial red area) were collected at the baseline and scheduled visits on Day 2, Weeks 1, 2, and 4 after the application of the cream. SCH objectively reflects the moisturizing effect of the cream on sensitive skin, while TEWL, pH, and sebum objectively reflect the repairing efficacy of the cream. EI, a* value, and facial red area objectively reflect its effect of alleviating erythema.

#### 2.4.3 TEWL

TEWL (g/m^2^/h) was assessed with a VapoMeter (Delfin Technologies, Finland). The index was measured 3 times in each target site, and the mean value was calculated.

#### 2.4.4 SCH

SCH was measured using a Corneometer (Courage & Khazaka Electronic GmbH, Germany) and presented in arbitrary units (a.u.). The index was measured 3 times in each target site, and the mean value was calculated.

#### 2.4.5 Sebum Secretion

The sebum secretion was determined by a Sebumeter (Courage & Khazaka Electronic GmbH, Germany). Each target site was measured once.

#### 2.4.6 Skin pH

The skin pH was determined by a skin‐pH‐meter (Courage & Khazaka Electronic GmbH, Germany). The index was measured 3 times in each target site, and the mean value was calculated.

#### 2.4.7 EI and a* Value

EI and a* value, which are the determinants of skin color, were detected using a Mexameter (Courage & Khazaka Electronic GmbH, Germany) and a Chromameter (Minolta Camera Co. Japan), respectively. Both indexes were measured 3 times in each target site, and the mean value was calculated.

#### 2.4.8 Image Analysis

VISIA‐CR (Canfield Scientific Inc., USA) was adopted to photograph the facial redness, and the analysis software was used to calculate the proportion of red areas.

#### 2.4.9 Self‐Assessment

Self‐assessment questionnaires about product efficacy were conducted by the subjects immediately after cream use and on Day 7, Week 2, and Week 4.

#### 2.4.10 Safety

Adverse events (AEs) were assessed and monitored throughout the study according to the Safety and Technical Standards for Cosmetics (2015 Version) approved by the National Medical Products Administration (NMPA) [27]. AEs were graded on a three‐point scale: mild = easily tolerated, moderate = difficult to tolerate, or severe = any event that prevents normal daily activities.

### 2.5 Statistical Analysis

All statistical analyses were conducted using a per‐protocol analysis with SPSS software (version 20.0, IBM International Business Machines Corporation). Only the subjects who completed trials were included in the statistical analyses. Data were presented as mean (±standard deviation [SD]) or median (interquartile range [IQR]) for continuous variables, and counts (%) for categorical variables, as appropriate. Changes from baseline were summarized as descriptive statistics and were compared using paired T‐test or Wilcoxon rank‐sum test. The severity of sensitive skin was classified according to the SS‐10 cut‐off scores [“sensitive skin” (SS‐10 global score ≥ 13), and “slightly sensitive skin” (13 > SS‐10 global score ≥ 5)] [28], and the Wilcoxon rank‐sum test was used for comparing the differences in skin physiological parameters and SS‐10 scores between two groups. All *p* values were adjusted for multiple comparisons using the Bonferroni correction. A *p* value < 0.05 with a 95% confidence interval (CI) was considered to be statistically significant.

## 3 Results

### 3.1 Demographics

A total of 92 healthy female subjects were recruited for the study, and ultimately 88 subjects completed the trial. Demographic characteristics of the subjects are summarized in Table 1. The mean age of the subjects was 40.7 ± 7.44 years. The phototype was mainly III (55.7%) and IV (43.2%). Regarding skin type, 50% (*n* = 44) had dry skin, 30.7% (*n* = 27) had normal skin, and 19.3% (*n* = 17) had combination‐to‐dry skin. At baseline, there were no significant differences between the sensitive and slightly sensitive subgroups in terms of demographics (*p* > 0.05). The mean SS‐10 score at baseline was 20.24 (±9.93). Based on the cut‐off scores of SS‐10 for sensitive skin, 68 (77.3%) subjects were classified as “sensitive skin” (global score ≥ 13), and 19 (21.6%) as “slightly sensitive skin” (global score ≥ 5 and < 13). Notably, one subject had a LAST score of 11 but an SS‐10 score of only 3, thus failing to meet the criteria for inclusion in any subgroup. The mean SS‐10 score was 23.62 ± 8.69 for the “sensitive” group and 9.05 ± 1.93 for the “slightly sensitive” population.

**TABLE 1:** Demographic characteristics.

Variables	Subjects (*n* = 88)
Age (years), mean ± SD	40.7 ± 7.44
Phototype, *n* (%)	
II	1, 1.1%
III	49, 55.7%
IV	38, 43.2%
Skin type, *n* (%)	
Dry	44, 50%
Normal	27, 30.7%
Combination‐to‐dry	17, 19.3%
SS‐10 global score, mean ± SD	20.24 ± 9.93
Severity of skin sensitivity	
Sensitive skin, *n* (%)	68, 77.3%
Slightly sensitive skin, *n* (%)	19, 21.6%

### 3.2 Sensitive Scale‐10

**FIGURE 1:**
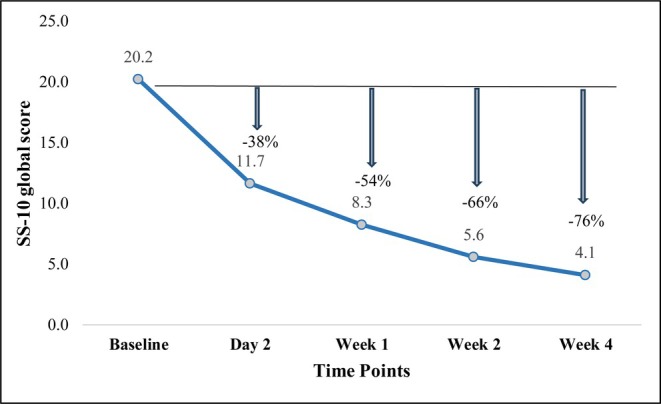
Change rate from baseline in SS‐10 global score at Day 2, Week 1, Week 2, and Week 4.

**FIGURE 2:**
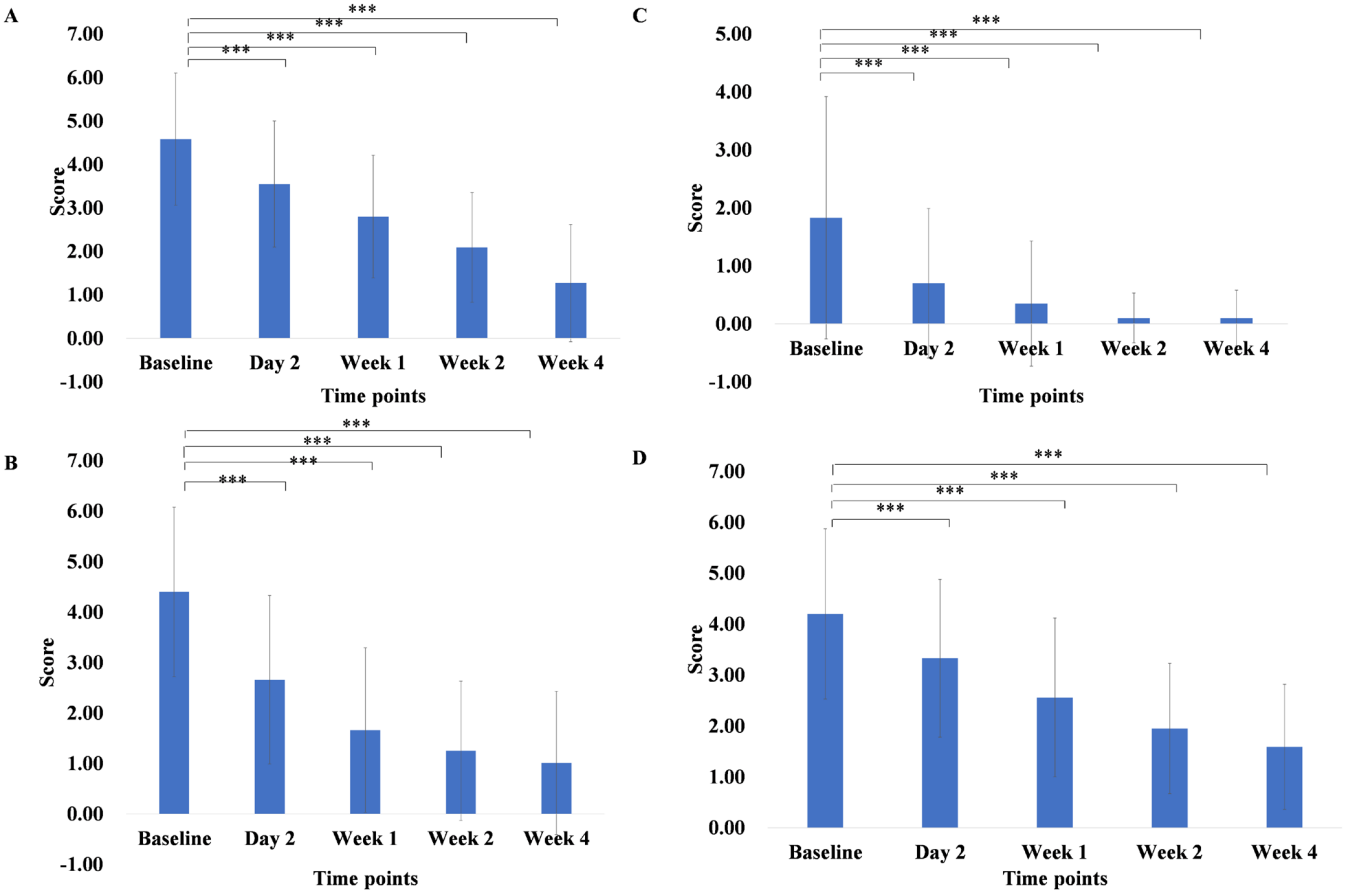
Score changes in skin irritation (A), tautness (B), itching (C), and redness (D) at baseline, Day 2, Week 1, Week 2, and Week 4. ***p < 0.001 versus baseline measurements, 95% CI.

The SS‐10 scores were assessed at baseline, Day 2, Weeks 1, 2, and 4 after cream use. The mean SS‐10 global score at baseline was 20.24 (±9.93), and dropped by 66% and 76% after 2 and 4 weeks of cream use, respectively (Figure 1). The mean scores of skin irritation, tightness, itching, and redness were reduced significantly after 4 weeks of cream application (*p* < 0.001) (Figure 2 and Table 2).

**TABLE 2:** Changes in items of SS‐10 before and after baseline.

Item scores of SS‐10	Baseline	Day 2	Week 1	Week 2	Week 4
Skin irritation	4.58 ± 1.52	3.55 ± 1.45	2.80 ± 1.41	2.09 ± 1.26	1.27 ± 1.35
*p*		0.000	0.000	0.000	0.000
Tightness	4.40 ± 1.68	2.66 ± 1.67	1.66 ± 1.63	1.25 ± 1.38	1.01 ± 1.42
*p*		0.000	0.000	0.000	0.000
Itching	1.83 ± 2.09	0.70 ± 1.29	0.35 ± 1.08	0.10 ± 0.43	0.10 ± 0.48
*p*		0.000	0.000	0.000	0.000
Redness	4.20 ± 1.67	3.33 ± 1.55	2.56 ± 1.56	1.95 ± 1.28	1.59 ± 1.23
*p*		0.000	0.000	0.000	0.000

### 3.3 Non‐Invasive Skin Physiological Parameters

The mean value of these skin physiological parameters before and after cream use is shown in Table 3. Throughout the 4‐week study period, there was an obvious increase in SCH at all time points (*p* < 0.001), which represented a great improvement in the skin surface hydration.

**TABLE 3:** Changes in skin physiological parameters at baseline and follow‐up visits.

Skin measurements	Baseline	Day 2	Week 1	Week 2	Week 4
SCH (AU)	57.52 ± 11.30	62.02 ± 11.45	65.46 ± 10.11	62.65 ± 12.36	72.76 ± 9.54
*p*		0.000	0.000	0.000	0.000
TEWL (g/h m^2^)	29.41 ± 16.02	27.99 ± 12.32	24.39 ± 7.69	24.98 ± 6.44	27.57 ± 9.45
*p*		0.148	0.001	0.005	0.255
Sebum level (μg/cm^2^)	33.58 ± 19.91	32.13 ± 22.84	33.93 ± 20.95	33.34 ± 20.01	38.75 ± 22.13
*p*		0.361	0.836	0.896	0.005
Skin pH (AU)	6.44 ± 0.74	6.43 ± 0.71	6.26 ± 0.73	5.84 ± 0.52	6.13 ± 0.68
*p*		0.883	0.006	0.000	0.000
EI (AU)	320.33 ± 55.01	302.75 ± 52.58	295.10 ± 55.47	293.73 ± 52.45	298.16 ± 53.59
*p*		0.000	0.000	0.000	0.000
a* value (AU)	12.20 ± 1.44	11.62 ± 1.37	11.38 ± 1.35	11.56 ± 1.28	11.68 ± 1.41
*p*		0.000	0.000	0.000	0.000
Facial red area (mm^2^)	344.15 ± 159.08	230.99 ± 149.20	196.18 ± 143.46	202.78 ± 133.81	199.00 ± 148.49
*p*		0.000	0.000	0.000	0.000

Abbreviations: EI, erythema index; SCH, stratum corneum hydration; TEWL, transepidermal water loss.

**FIGURE 3:**
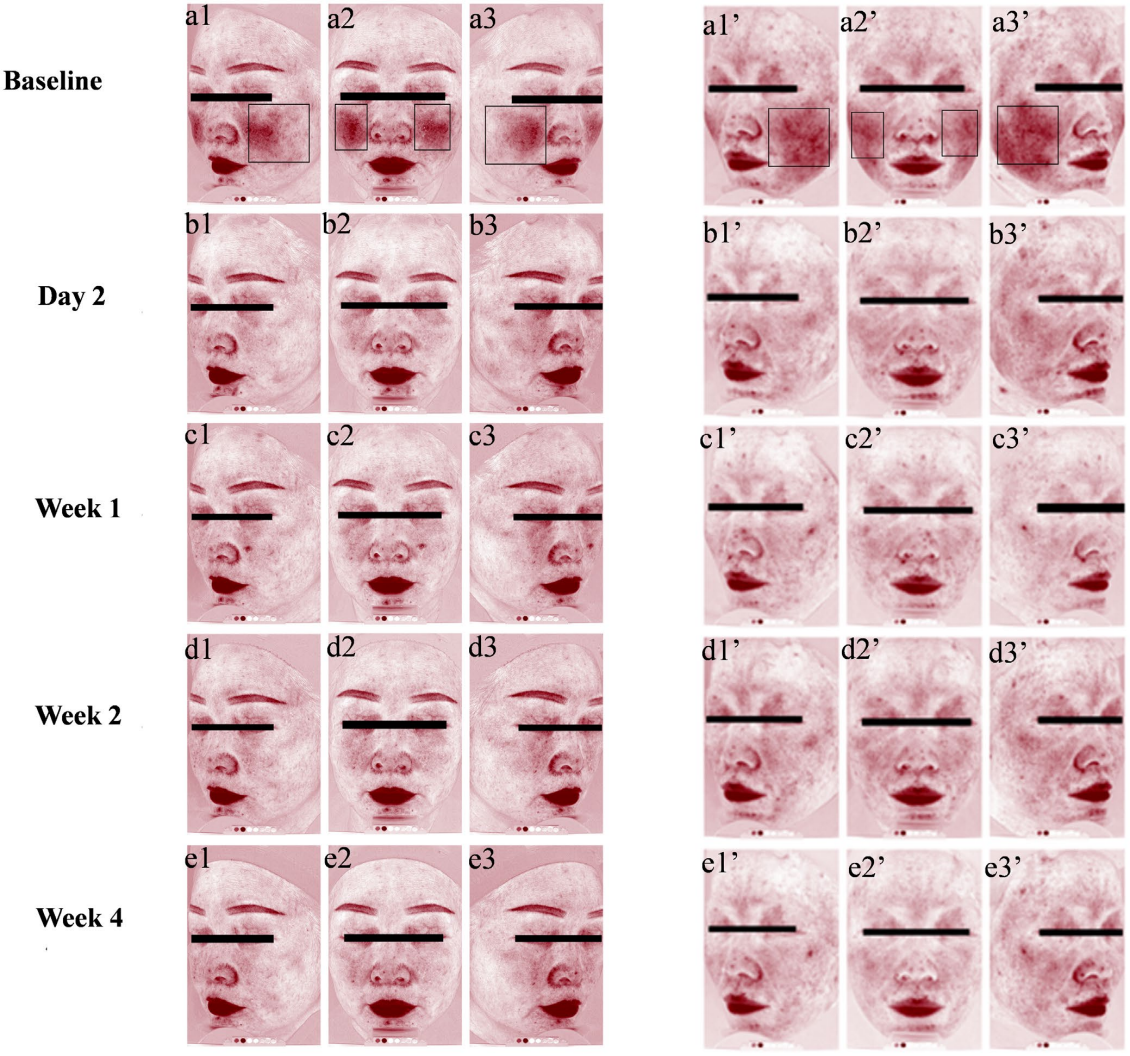
Images of the red area taken by VISIA. (a: 1–3, 1′–3′): at baseline; (b: 1–3, 1′–3′): at Day 2; (c: 1–3, 1′–3′): at Week 1; (d: 1–3, 1′–3′): at Week 2; (e: 1–3, 1′–3′): at Week 4.

TEWL decreased significantly during the first week (24.39 ± 7.69) and the second week (24.98 ± 6.44) of cream use (*p* < 0.01). Sebum secretion levels also gradually increased with the use of cream, significantly rising to 38.75 (±22.13) by the fourth week (*p* < 0.01). After applying the cream, the skin pH decreased significantly at Week 1 (*p* < 0.01), and Weeks 2 and 4 (*p* < 0.001). The significant decrease of EI, a* value, and red area was maintained at all time points throughout the 4‐week study period (*p* < 0.001). VISIA images further corroborated visible improvements in facial erythema (Figure 3).

### 3.4 Self‐Assessment Questionnaire (Subjects' Satisfaction)

**FIGURE 4:**
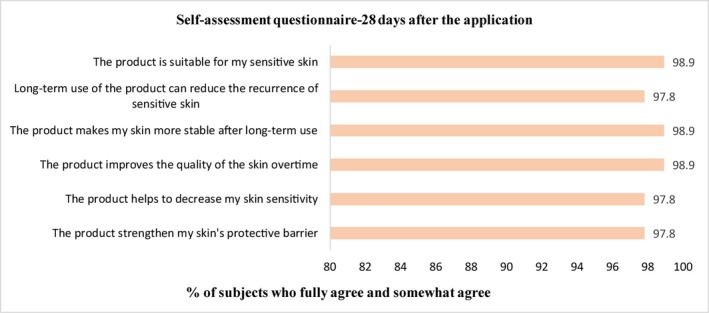
Subjects' satisfaction immediately and after 28 days of continued daily use of the facial cream.

Detailed results of subjects' satisfaction immediately and 28 days after using the facial cream were presented in Figure 4. Most of the subjects agreed that the product could immediately relieve skin irritation, improve facial redness, and reduce skin discomfort after the first use. After 28 days of use, most subjects believed that the product could reduce skin sensitivity, strengthen the skin barrier, and make the skin status more stable. All subjects indicated a willingness to continue using this product.

### 3.5 Safety

Six possibly product‐related AEs were reported during the study period, mainly manifesting as acne (new papules and comedones) on the cheeks, jaw, or around the lips. Only one subject reported skin tingling during the product usage period. These reactions resolved spontaneously within a week without special treatment. No serious adverse events (SAEs) were observed throughout the study (Table S1).

### 3.6 Subgroups According to SS‐10 Score

**FIGURE 5:**
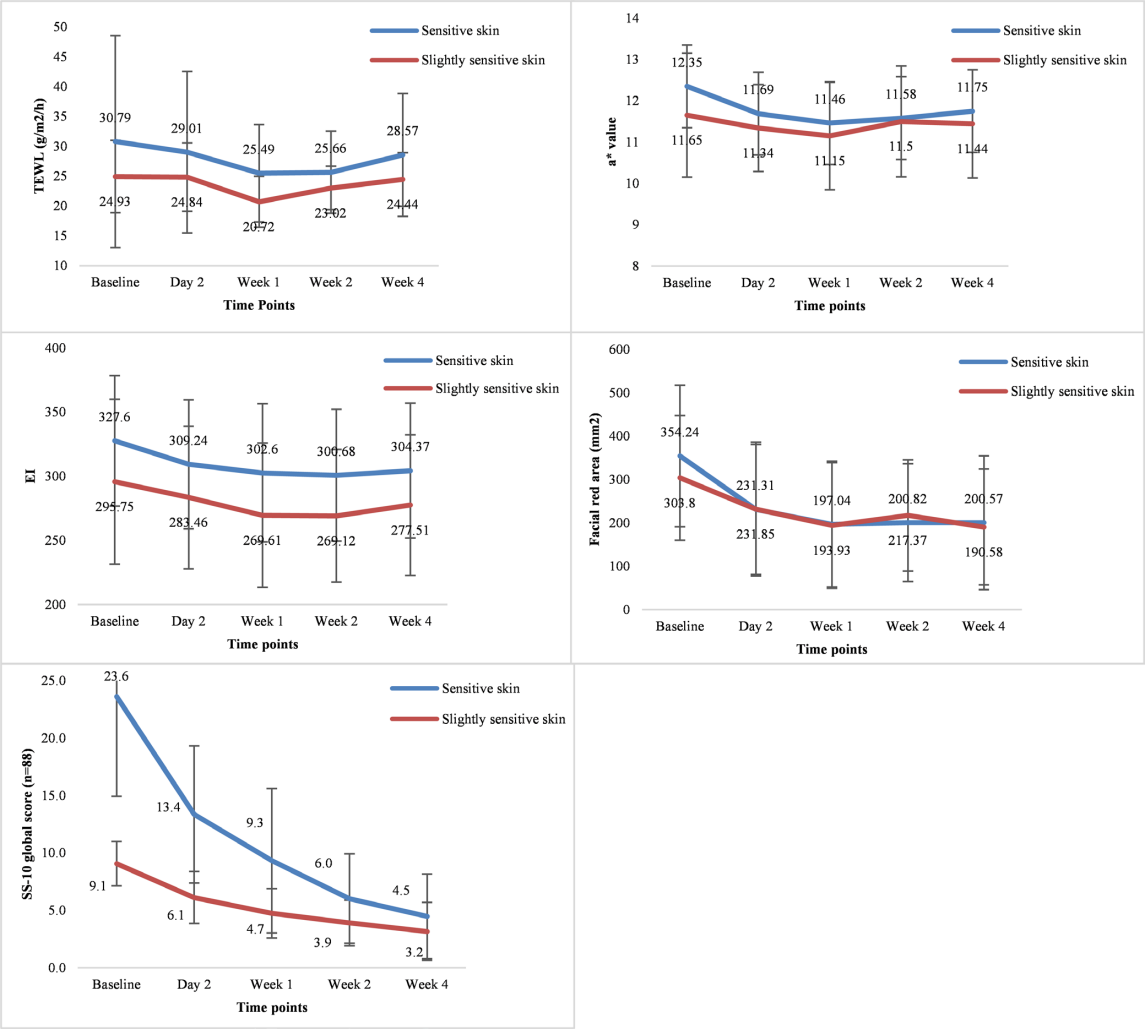
Changes in transepidermal water loss (TEWL) (A), erythema index (EI) (B), a* value (C), facial red area (D), and SS‐10 score (F) between the sensitive skin (*n* = 68) and slightly sensitive skin (*n* = 19) at baseline and different study visits.

The severity of sensitive skin was classified into sensitive skin (*n* = 68) and slightly sensitive skin (*n* = 19) according to the defined SS‐10 cut‐off scores, and the improvement of skin physiological parameters was evaluated and compared between the two groups. The difference of SS‐10 scores in the sensitive skin group was significantly higher than those in the slightly sensitive group at Day 2 (*p* < 0.001), Week 1 (*p* < 0.001), Week 2 (*p* < 0.001), and Week 4 (*p* < 0.001), indicating significant improvement in the SS‐10 scores of the sensitive skin group. In the comparison of physiological parameters between the two groups, the difference of a* value in the sensitive skin group was significantly higher than that of the slightly sensitive skin group at Week 2 (*p* < 0.05). However, no significant differences were found in the differences of TEWL, EI, a* value (except at Week 2), and facial red area (*p* > 0.05). Nevertheless, the overall improvement in physiological parameters in the sensitive skin group was numerically superior to that in the slightly sensitive group (Figure 5 and Table 4).

**TABLE 4:** Comparision of the differences in skin physiological parameters and SS‐10 scores between sensitive skin and slightly sensitive skin group.

Skin measurements		Day 2	Week 1	Week 2	Week 4
TEWL (g/h m^2^)	Sensitive skin group	1.78 ± 10.08	5.30 ± 15.83	5.13 ± 16.07	2.22 ± 16.94
	Slightly sensitive group	0.08 ± 4.61	4.21 ± 3.97	1.91 ± 4.65	0.48 ± 4.34
	*p*	0.548	0.569	0.558	0.943
EI (AU)	Sensitive skin group	18.36 ± 30.89	25.00 ± 31.18	26.93 ± 31.93	23.23 ± 29.85
	Slightly sensitive group	12.30 ± 42.13	26.14 ± 39.40	26.63 ± 40.16	18.25 ± 42.26
	*p*	0.444	0.861	0.930	0.947
a* value (AU)	Sensitive skin group	0.66 ± 1.23	0.89 ± 1.15	0.77 ± 1.22	0.60 ± 1.16
	Slightly sensitive group	0.31 ± 1.03	0.51 ± 1.22	0.15 ± 1.00	0.22 ± 0.89
	*p*	0.227	0.180	0.015	0.131
Facial red area (mm^2^)	Sensitive skin group	122.93 ± 212.73	157.21 ± 219.75	153.42 ± 223.33	153.67 ± 188.79
	Slightly sensitive group	71.95 ± 184.60	109.87 ± 161.31	86.43 ± 155.44	113.22 ± 189.49
	*p*	0.233	0.225	0.098	0.299
	Mean difference (95% CI)	111.80 [67.49, 157.54]	146.87[102.27, 193.10]	138.79 [97.00, 185.30]	144.84 [104.77, 182.10]
SS‐10	Sensitive skin group	10.26 ± 8.54	14.29 ± 9.96	17.60 ± 8.82	19.15 ± 9.09
	Slightly sensitive group	2.95 ± 2.39	4.32 ± 2.65	5.16 ± 2.50	5.89 ± 3.05
	*p*	0.000	0.000	0.000	0.000

*Note:* Difference was defined as the post‐product usage measurement value minus the baseline measurement value.

Abbreviations: EI, erythema index; SCH, stratum corneum hydration; TEWL, transepidermal water loss.

## 4 Discussion

For severely sensitive skin, topical and systemic medications can be used, but they carry the risk of side effects. For most people with sensitive skin, scientific daily skin care is a more essential measure to manage sensitive skin, and choosing an appropriate and safe skin care product is the key step. Based on the subjective assessment of physical signs and objective measurement of skin physiological parameters, the use of the investigated cream effectively strengthened the skin barrier and improved various manifestations of sensitive skin, such as reducing skin dryness, relieving skin sensitivity, and rapidly alleviating facial redness. These findings are mostly consistent with the results of previous efficacy tests.

Cetaphil soothing and comforting facial cream is a product developed for dry, sensitive skin, containing *Centella asiatica* leaf extract, ceramide NP, and panthenol. Previous studies have indicated that extracts of *Centella asiatica* , ceramides, and panthenol are all active ingredients beneficial for sensitive skin [21]. The essence of 5% nanoemulsion loaded with paeonol and madecassoside significantly reduced TEWL, a* values, erythropoietin, the amount of non‐inflammatory acne, and the amount of inflammatory acne on the facial skin of individuals with sensitive skin, while increasing SCH. Additionally, it increased sebum content in dry skin and decreased sebum secretion in oily skin [29]. Hiroshi Nojiri et al. found that topical application of a ceramide complex cream significantly increased the total natural ceramide levels in the stratum corneum, improved the ceramide profile, further increased SCH, decreased TEWL, and effectively ameliorated lactic acid sensations in sensitive skin [22]. Additionally, Xianghua Zhang et al. reported that a facial mask enriched panthenol also containing *Centella asiatica* extract and ceramide effectively regulated sebum production improved SCH, reduced TEWL, and diminished skin redness across dry and oily sensitive skin [30]. However, few studies have been reported on the combined effects of the three ingredients on sensitive skin.

This single‐center, prospective, and self‐controlled study evaluated the efficacy and safety of Cetaphil soothing and comforting facial cream for relieving and repairing dry sensitive skin through professional researcher evaluation, skin physiological index measurements, and subject self‐assessment questionnaires involving 88 subjects. The results suggested that the product effectively alleviated symptoms of sensitive skin such as irritation, tautness, and itching. A significant reduction in TEWL was observed at Weeks 1 and 2 (*p* < 0.01), along with decreased skin pH at Week 1 (*p* < 0.01), and Weeks 2, 4 (*p* < 0.001). Additionally, sebum production was markedly increased at Week 4 (*p* < 0.01), while SCH showed significant improvement at all time points (*p* < 0.001). These were mostly in agreement with findings from previous studies mentioned above [22, 29, 30]. Subgroup analysis based on SS‐10 cut‐off values revealed a more pronounced improvement in the sensitive skin group compared to the slightly sensitive skin group.

During the trial, a total of 6 subjects experienced AEs such as pimples and comedones, and all were resolved within 1 week. Based on an assessment by the professional dermatologist, the six AEs were judged to be mild and possibly related to the product. Only one subject reported skin tingling, a symptom commonly associated with sensitive skin, during the product usage period, which resolved spontaneously within 2–3 min. Therefore, the dermatologist concluded that the AEs during the trial were all mild, indicating a certain level of safety for this product.

As the primary endpoint, the mean SS‐10 global score showed a significant reduction of 76%, indicating a decrease in skin sensitivity. More specifically, the symptoms of skin irritation, tightness, itching, and redness were relieved. This improvement reflected a substantial enhancement in comfort and well‐being for the patients, thereby improving patients' quality of life. Also, it showed that SS‐10 could be used to track the changing process to adjust the subsequent regimen [1, 11].

Currently, there exist two primary disputes regarding the cut‐off values for classifying the severity of sensitive skin: one utilizes 5 and 13 points as the thresholds for slightly sensitive and sensitive skin, respectively, while the other adopts 20 and 60 points. The “Clinical Guidelines for the Diagnosis and Treatment of Sensitive Skin in China (2024 Edition)” [31] recommends the former, whereas some surveys in Hong Kong adopt the latter. However, the latter approach tends to overlook patients with milder symptoms, leading to an underestimation of the prevalence rate, and it does not exclude patients with other skin diseases. Our study investigated subjects who self‐reported sensitive skin without other skin diseases, consistent with the study population of Legeas et al. [28], and more aligned with the definition of sensitive skin by the International Forum for the Study of Itch (IFSI). Therefore, our study has decided to adopt the following criteria for classifying the severity of sensitive skin: a total SS‐10 score of ≥ 13 points is considered “sensitive skin,” while a score between 5 and 13 points is designated as “slightly sensitive skin.” Grouping based on SS‐10 and cut‐off values, the condition was more severe in subjects in the sensitive skin subgroup, suggesting that the SS‐10 assessment and the defined cut‐off value have certain clinical significance in the Chinese population [11, 28]. Among the subjects with slightly sensitive skin, although there was relatively limited room for improvement, the results of the study also showed a good performance after the cream application.

The measurement of non‐invasive skin physiological parameters is of great significance for evaluating the efficacy of the investigated product [8, 16]. It is well known that sensitive skin has a barrier dysfunction problem, which is characterized by an increased TEWL and decreased SCH. As one of the important ingredients, ceramide is considered to restore intercellular lipid content, which may help maintain stratum corneum integrity. Panthenol has been shown to penetrate the stratum corneum easily and play a good moisturizing role, keeping the skin soft and pliable. Many studies have suggested that skin care products containing panthenol and ceramides can strengthen skin barrier function in the short term by reducing TEWL and increasing SCH [21, 32, 33]. This is mostly consistent with our findings, which showed significant changes in TEWL and SCH after only 2 days of the cream use in sensitive skin subjects.

Sensitive skin patients usually present with very dry skin and low sebum levels, resulting in a disturbance of the barrier function of the skin [34]. In this study, half of the subjects had dry skin; however, the sebum secretion was significantly increased until the fourth week of cream use. A certain degree of balance between hydration and sebum levels played a critical role in protecting skin integrity and providing smooth skin texture [33, 35].

The acid mantle is known to be essential for the maintenance of stratum corneum homeostasis, skin barrier permeability, and anti‐microbial protection [36, 37, 38]. Elevated skin surface pH has been reported in subjects with sensitive skin, atopic dermatitis, acne vulgaris, or other skin disorders [36, 38, 39, 40]. Expert consensus on holistic skin care routine [17] also recommends the use of skin care products with mildly acidic pH, which can help maintain the relatively acidic environment on the skin surface. After the use of this cream, the skin pH of the subjects gradually decreased and stayed between 5.8 to 6.3, while TEWL also showed a downward trend, which may indicate that the regulation of pH has a positive significance for the enhancement of skin barrier function [41].

Subjects with sensitive skin are prone to erythema, which might be caused by microcirculation changes and inflammation [42, 43]. There was a significant decrease in a* values, EI, and facial red area in sensitive skin after only 2 days of the cream use, indicating the rapid suppression of the skin inflammation. We hypothesize that this may be related to *Centella asiatica* extract and panthenol in the formula. Studies described that the saponins, flavonoids, and phenolic acids in *Centella asiatica* extract could reduce the expression of TRPV1 [20], inhibit the release of inflammatory mediators (such as TNF‐α, interleukin‐1b/IL‐1b, IL‐4, IL‐13), the activity of iNOS and COX‐2, and the activation of the NF‐κB pathway, as well as reduce the infiltration of mast cells and other inflammatory cells [44, 45, 46, 47]. An in vitro study also showed that panthenol was able to inhibit the release of inflammatory mediators (such as prostaglandin E2, IL‐6, and thymic stromal lymphopoietin levels) [48]. In addition, *Centella asiatica* extract has been reported to improve microcirculation by enhancing vascular endothelial cell integrity, reducing capillary filtration rate, and modulating vascular wall metabolism [45, 46].

This study is a single‐center, self‐controlled trial with the following limitations: Firstly, the trial was conducted exclusively among Chinese healthy females and did not involve other race/ethnic groups, males, and those with comorbidities. These limits may affect the generality and reproducibility of the results. Secondly, subjective outcomes may be influenced by self‐reporting bias. The lack of blinding in the subjective assessment of the investigator may also introduce subjective bias. Thus, the accuracy of the results could be improved in the future by increasing the number of investigators for simultaneous evaluation and expanding the sample size. Thirdly, the discrepancy in the number of subjects between the two sensitive skin subgroups could lead to deviations in intergroup comparisons. Finally, the lack of a placebo group makes it impossible to rule out the effects of ingredients other than the three active ingredients in the product, *Centella asiatica* leaf extract, ceramide NP, and panthenol, or to determine the difference between the product effect and natural variation, so the addition of a placebo group would have made the results more compelling and convincing. Future studies should expand the sample size, balance the number of participants in each group, and conduct multi‐center, randomized controlled, and blinded studies with the addition of a placebo group to more accurately and comprehensively evaluate the product's effectiveness in improving the clinical characteristics of sensitive skin.

## 5 Conclusions

In summary, this single‐center, prospective, and self‐controlled study indicated the short and long‐term effectiveness and safety profile of the facial cream. The investigated product improved the facial redness of sensitive skin in a short duration, regulated the balance of skin hydration and sebum level, and provided a favorable soothing, and comforting effect for individuals with sensitive skin. This not only provides dermatologists with new ideas for treating sensitive skin but also allows consumers to effectively alleviate the symptoms caused by sensitive skin and enhance their user experience. As an essential part of the skin care routine, this specific moisturizer provides a new option for people with sensitive skin.

## Author Contributions

Z.S.: methodology, essay revision, graphing, and data curation; Y.Z.: writing – reviewing, discussing, and editing; J.Y.: methodology; W.L.: supervision and investigation; C.Y.: project administration and writing.

## Ethics Statement

The study was conducted according to the guidelines of the Declaration of Helsinki, and approved by Use Committees (IACUC) of the Third Affiliated Hospital of Sun Yat‐Sen University. (approval code [2020]008‐03).

## Consent

Informed consent was obtained from all patients/participants involved in the study.

## Conflicts of Interest

The authors declare no conflicts of interest.

## Supporting information


**Figure S1.** Sensitive Scale‐10.
**Table S1.** Adverse events (AEs) reported during the trial period.

## Data Availability

The data that support the findings of this study are available from the corresponding author upon reasonable request.
