# Supplementary material for: The Effectiveness and Safety of a Skin Care Product With Centella asiatica Leaf Extract, Ceramide NP, and Panthenol in Subjects With Sensitive Skin: A Prospective, Observational Study
Source: J Cosmet Dermatol. 2025 Jul 19;24(7):e70324. doi: 10.1111/jocd.70324 (PMC12274965; doi:10.1111/jocd.70324)
Supplement: Supplementary file 1 — Figure S1. Sensitive Scale‐10. Table S1. Adverse events (AEs) reported during the trial period. [file JOCD-24-e70324-s001.docx]

**Supplementary materials**

**Figure S1** Sensitive Scale-10

**DEGREE OF OVERALL SKIN IRRITATION DURING THE PAST 3 DAYS**

Using a vertical line, indicate the symptoms felt during the past 3 days on the horizontal

line (0 = absence of irritation, 10 = intolerable irritation)

Skin irritation

0

Min

10

Max

**SEVERITY OF SKIN CONDITION DURING THE PAST 3 DAYS**

Please indicate the intensity of each of the following symptoms during the past 3 days.

0 = zero intensity, 10 = intolerable intensity): darken one number between 0 and 10.

| Skin condition felt: | |
| --- | --- |
| Tingling | 🄋 ➀ ➁ ➂ ➃ ➄ ➅ ➆ ➇ ➈ ➉ |
| Burning | 🄋 ➀ ➁ ➂ ➃ ➄ ➅ ➆ ➇ ➈ ➉ |
| Sensations of heat | 🄋 ➀ ➁ ➂ ➃ ➄ ➅ ➆ ➇ ➈ ➉ |
| Tautness | 🄋 ➀ ➁ ➂ ➃ ➄ ➅ ➆ ➇ ➈ ➉ |
| Itching | 🄋 ➀ ➁ ➂ ➃ ➄ ➅ ➆ ➇ ➈ ➉ |
| Pain | 🄋 ➀ ➁ ➂ ➃ ➄ ➅ ➆ ➇ ➈ ➉ |
| General discomfort | 🄋 ➀ ➁ ➂ ➃ ➄ ➅ ➆ ➇ ➈ ➉ |
| Hot flashes | 🄋 ➀ ➁ ➂ ➃ ➄ ➅ ➆ ➇ ➈ ➉ |
|  |  |
| Visible skin conditions: | |
| Redness | 🄋 ➀ ➁ ➂ ➃ ➄ ➅ ➆ ➇ ➈ ➉ |

**Table S1** Adverse Events (AEs) Reported During the Trial Period

| **Sub** | **Reaction Onset Time** | **Reaction Site** | **Symptoms** | **Severity** | **Duration** | **Management** | **Outcome** |
| --- | --- | --- | --- | --- | --- | --- | --- |
| 15 | July 21, 2023 | Face (mainly cheeks and forehead) | Six new papules | Mild | About 1 week | None | Symptoms spontaneously subsided |
| 28 | July 18, 2023 | Cheeks | Tingling sensation | Mild | 2 - 3 minutes | None | Symptoms spontaneously subsided |
| 29 | July 24, 2023 | Face (mainly cheeks and forehead) | 10 - 12 new comedones | Mild | 3 - 4 days | None | Symptoms spontaneously subsided |
| 42 | September 18, 2023 | Left cheek | Two new papules, four new comedones | Mild | 3 - 4 days | None | Symptoms spontaneously subsided |
| 62 | September 22, 2023 | Jaw | One new papule | Mild | 3 days | None | Symptoms spontaneously subsided |
| 83 | September 22, 2023 | Jaw, perioral area | 12 - 13 new comedones | Mild | 2 - 3 days | None | Symptoms spontaneously subsided |
